# Are physicians required during HEMS winch rescue missions?

**DOI:** 10.1186/cc12220

**Published:** 2013-03-19

**Authors:** PB Sherren, C Hayes-Bradley, C Reid, B Burns, K Habig

**Affiliations:** 1 Greater Sydney Area HEMS, Sydney, Australia

## Introduction

A winch-capable helicopter emergency medical service (HEMS) offers several advantages over standard rescue operations. Little is known about the benefit of physician winching in addition to a highly trained paramedic. We analysed the mission profiles and interventions performed during rescues involving the winching of a physician in the Greater Sydney Area HEMS (GSA-HEMS).

## Methods

All winch missions involving a physician from August 2009 to January 2012 were identified from the prospectively completed GSA-HEMS electronic database. A structured case-sheet review for a predetermined list of demographic data and physician-only interventions (POI) was conducted.

## Results

We identified 130 missions involving the winching of a physician, of which 120 case sheets were available for analysis. The majority of patients were traumatically injured (90%) and male (85%) with a median age of 37 years. Seven patients were pronounced life extinct on the scene. A total of 63 POI were performed on 48 patients. Administration of advanced analgesia was the most common POI making up 68.3% of interventions. Patients with abnormal RTSc^2 ^scores were more likely to receive a POI when compared with those with normal RTSc^2 ^(*P *= 0.03). The performance of POI had no effect on median scene times (45 vs. 43 minutes; *P *= 0.51). See Tables 1 and 2.

## Conclusion

Our high POI rate of 40% coupled with long rescue times and the occasional severe injuries supports the argument for winching doctors. Not doing so would deny a significant proportion of patients time-critical interventions, advanced analgesia and procedural sedation.

**Table 1:** Demographic data, timings and Coded Revised Trauma Score (RTSc^2^)

	Total (*n *= 120)
Male	102 (85)
Median (IQR) age (years)	37 (26 to 53)
Received a physician-only intervention	48 (40)
Pronounced life extinct on arrival	7 (5.8)
RTSc^2^	
7.8408	104 (86.7)
7.0001 to 7.8407	2 (1.7)
6.0001 to 7.0000	4 (3.3)
5.0001 to 6.0000	4 (3.3)
4.0001 to 5.0000	0 (0)
<4.0001	6 (5)

**Table 2:** Interventions performed

Physician-only intervention	Number of interventions (*n *= 63)
Analgesia/procedural sedation	
Intravenous ketamine	42 (66.7)
Intravenous fentanyl	1 (1.6)
Fascia iliaca compartment block	1 (1.6)
Airway management	
Rapid sequence induction and intubation	4 (6.3)
Surgical airway	1 (1.6)
Circulatory support	
Adult intraosseous access	1 (1.6)
Blood transfusion	2 (3.2)
Orthopaedic manipulation of joint/limb	6 (9.5)
Thoracostomy	1 (1.6)
Diagnostic ultrasound	1 (1.6)
Hypertonic saline administration	3 (4.8)

Data presented as *n *(%).

